# Patients’ perspective of attending nursing consultations—A pilot and feasibility study

**DOI:** 10.1002/nop2.522

**Published:** 2020-06-19

**Authors:** Jytte Graarup, Ida Elisabeth Højskov

**Affiliations:** ^1^ Department of Cardiothoracic Surgery The Centre for Cardiac, Vascular‐ Pulmonary and Infectious Diseases Rigshospitalet University of Copenhagen Copenhagen Denmark

**Keywords:** nursing intervention, nursing practice, patients’ experience, self‐management

## Abstract

**Aim:**

The aims were to explore: (a) how patients with advanced heart and lung failure accept the overall framework of the nursing consultations and (b) the patients’ acceptability and applicability of the nursing consultations.

**Design:**

Qualitative study.

**Methods:**

Interviews were conducted in an holistic frame and analysed using Graneheim and Lundman's qualitative content thematic analysis. Patients were interviewed between April and May 2018 regarding their general view of the nursing consultation and their experience of the framework inspired by R. R. Parse.

**Results:**

The overall theme was A confidential moment with the nurse to deal with and become more aware of what is important, based on following subthemes: “An option that makes sense,” “Scheduled time with the nurse is important” and “To find a new normality in everyday life.” The framework addressed a space of freedom requested by the informants, for whom attending nursing consultations was useful and meaningful, enabling them to reflect on everyday challenges.

## 1 INTRODUCTION

Patients with advanced heart and lung failure experience symptoms and problems related to their treatment and the underlying disease (van Houtum, Rijken, & Groenewegen, 2015). These symptoms and problems provide useful and important knowledge for healthcare professionals (HCP) to understand the patient's situation and to schedule an individual course of treatment and care. Clinical practice and research apply different terms describing cooperation between the patient and HCP’s for instance: patient involvement, patient participation and patient self‐care management. The different terms are used as presented in the context of the applied studies and in respect for these.

## 2 BACKGROUND

The European guidelines on cardiovascular disease prevention in clinical practice (Members: et al., 2016), supplemented by national‐(Health n.d.) and local guidelines on patient involvement (Rigshospitalet n.d.), recommend patient/relatives’ participation to support patient's self‐management to achieve better quality of life and add years of life. Early everyday life after lung transplantation is a balance between joy and challenges (Graarup, Mogensen, Missel, & Berg, 2017). The patients experienced an overwhelming postoperative improvement in physical function, but felt fragile because of the physical and psychological challenges (Graarup et al., 2017). Furthermore, they struggled with medical side effects and had difficulties adhering to requirements of life post‐transplant and problems in being compliant regarding medication (Graarup et al., 2017).

Like patients with lung failure, patients with heart failure also experience challenges in everyday life, which are related to both the chronic disease and to handling everyday life. Being chronically ill means to handle both physical and mental challenges and to strive to follow medical regimes. These findings are supported by similar findings in other studies of patients with chronic diseases (Martin, Stone, Scott, & Brashers, 2010) (Ivarsson, Ekmehag, & Sjoberg, 2013) (Singer et al., 2013) (Dew et al., 2015). Therefore, handling everyday life requires knowledge about the disease and competencies in self‐management for the patients to be able to make independent decision supplemented by capability related to life in general (Ontario, 2013) (Dawson‐Rose et al., 2016).

It is important for patients to protect the family from disease‐related worries. Therefore, it can be desirable for patients to have the opportunity to discuss and reflect on their challenges in a familiar relation with a HCP (Rees & Williams, 2009). To address the patients’ need for a dialogue, scheduled nursing consultations as an intervention are suggested (Graarup et al., 2017). Previous studies have explored the interaction and communication between patients and nurses (Barratt & Thomas, 2018a, 2018b; Defibaugh, 2017); however, the perspective of this study has been to elaborate patients’ need for nursing consultations with focus on everyday worries.

Patient participation in the patient–practitioner encounter is a key factor in influencing quality of life (Rees & Williams, 2009). Patients' self‐care management involves psychosocial as well as medical management. HCP need to recognize and value patients' views and experiences to support their self‐care management to improve medication applicability and disease awareness. It is evident that patients with chronic diseases need competencies in self‐care to increase their capacity to think critically and make informed decisions about their health (Kambhampati, Ashvetiya, Stone, Blumenthal, & Martin, 2016). Thinking in the long‐term nurses must understand the importance of promoting patient self‐management if they work with patients in the self‐management of long‐term conditions (Wilkinson, Whitehead, & Crowe, 2016). Research in the field of outpatient clinics with separate nursing consultations has shown that nurses have the competences to conduct individual consultations regarding everyday life with a chronic disease (Robb, Fleming, & Dietert, 2002).

Before the nursing consultation could be implemented in clinical practice, it was necessary to ensure that the proposed theoretical framework was useable for all affiliated groups of patients with advanced heart and lung failure. The Human Becoming Practice Methodology by Rosemarie R. Parse was chosen as an inspiration for the theoretical framework for the nursing consultation, because of the patient‐centred approach and the opportunity for the patient to bring up and reflect on actual topics of importance (Parse, 1998). Parse is, among others, inspired by the German philosopher Martin Heidegger's work and ideas on the phenomenological and hermeneutic understanding of human beings (Heidegger, 2014). Heidegger's philosophy is ontologically concerned with the question of “being,” of what it means for something to be, namely human beings. Analogously to this, Parse has introduced the concept Humanbecoming. The approach is based on an holistic patient view and focuses on the handling of life and managing the treatment. The topics dealt with initially cover life before the disease, present life with the disease and visions of future short‐ and long‐term life. An inspirational guide can form the basis for the nursing consultations. The guide consists of several elements and issues (medical, psychosocial and educational).

During the conversation, it is possible to imagine possibilities inspired by three dimensions of the theory of Parse described in her book: *The Human Becoming School of* Thought: A perspective for Nurses and Other Health Professionals (Parse, 1998). According to this, there are three overall ways to change health: (a) creative imagining, which is to see, hear and feel what a situation might be like if lived in a different way; (b) affirming personal becoming, that is thinking critically about one's personal patterns, preferences and values and changing one's attitude to change health; and (c) glimpsing the paradoxical, looking at incongruence in a situation, changes the view often held. These steps must be facilitated through dialogue and give meaning to the past, present and future, discussing events and possibilities and moving along with envisioned possibilities to create correlation in time. A nurse is present in the dialogue through discussions, silent immersion and shared reflections, allowing the patient time and space to explore, discover and find answers and solutions to their own health beliefs.

In this study, the opening question of the nursing consultation was: “How has your heart/ lung disease affected your everyday life?”. The conversation followed the themes chosen by the patient but reflected by the nurse. The questions asked were inspired by Parses’ Humanbecoming Practice Methodology where she states: “health can only be described by the person who lives and is the process of becoming one” (Parse, 1998).

As the chosen theoretical framework has never previously been used in the department, this pilot and feasibility study was conducted to explore: whether it is feasible to open the conversation, needed by the patient in this specific context, using this theoretical framework? The aims of the pilot and feasibility study were to explore: (a) how patients with advanced heart and lung failure accept the overall framework of the nursing consultations and (b) the patients’ acceptability and applicability of the nursing consultations. In other words, the purpose was to gather the patient's immediate thoughts about this nursing consultation set‐up.

## 3 DESIGN

The study adopted a qualitative approach designed in an holistic frame inspired by Parse.

An holistic approach offers a frame to understand and explain the meaning of experience by interpreting interviews transcribed as a text. The method of analysis was Graneheim and Lundman qualitative content thematic analysis. Reporting of this study followed the criteria for reporting qualitative research, COREQ guidelines, FileS1.

## 4 METHOD

Nursing consultations were offered to participants of this study to explore how the framework, acceptability and applicability of the nursing consultation were experienced. The informants were recruited from patients who visited the outpatient clinic for the annual follow‐up. To represent the group of patients with advanced heart and lung failure, the patients were approached according to art of disease and treatment. Everyone invited accepted the invitation and were offered a face‐to‐face nursing consultation.

Thirteen patients between the ages of 20–71 years took part in interviews, reflecting on the nursing consultation, held at the Department of Cardiology, Copenhagen University Hospital, in April and May 2018. The nursing consultation was scheduled, duration half an hour, in a room prepared for an undisturbed conversation. The overall purpose of the nursing consultation was to give the informants an opportunity to talk about topics of importance with the nurse, in this case one of the authors, trained in The Humanbecoming Practice Methodology. The eligible participants were more than 18 years old, spoke Danish, were not suffering from dementia and were asked consecutively. The patients were asked to talk broadly about the nursing consultation. The interviews lasted between 2–5 min and were taped and the text transcribed to ensure consistency.

### 4.1 Data analysis

The interviews were analysed by Graneheim and Lundman's qualitative content thematic approaches (Graneheim & Lundman, 2004), which include the aim to obtain credibility, dependability and transferability when analysing the text. Thereby the analysis method was chosen.

#### 4.1.1 Credibility

Credibility refers to how well data and the process of analysis address the intended focus. First off all, it is important to choose the focus, the participants and how to collect data. After collecting data, it is important to select suitable meaning units and to deal with how well categories and themes cover data. Credibility is also about how to judge between differences and similarities. To approach this is to show representative quotations from the transcribed text.

#### 4.1.2 Dependability

Dependability deals with the degree to which data change over time and alterations are made in the researcher's decisions during the analysis process. When data are collected over time, there is a risk of inconsistency as interviewing and observing is an evolving process. An open dialogue in the research team can strengthen the extent to which judgement about similarities and differences of content are consistent over time.

#### 4.1.3 Transferability

Transferability refers to the extent to which the finding of the study can be transferred to other groups of patients. A solid presentation of the findings with appropriate quotations will enhance transferability.

Data analysis was carried out by the authors who carefully read each interview text several times to find sentences with content areas revealing: (a) How did the informants experience the opportunity to attend a nursing consultation? and (b) what was the informants’ experience of the overall framework and setting?”

The aim of the analysis was to identify themes which describe the informants’ experiences of the phenomena and mark them as “meaning units.” As a result, parts of the interview text were highlighted and listed as a “meaning unit.” Subsequently, “meaning units” were reduced to “condensed meaning units,” keeping the message of the text, and finally, “condensed meaning units” were marked as “subthemes.” “Subthemes” provided an overview of the findings of the analysis by maintaining the message of each “condensed meaning unit." The researchers went through the whole analysis process individually before they met, compared and discussed the themes.

## 5 ETHICAL CONSIDERATIONS

Confidentially procedures were followed, and all patients participated on an informed, independent and voluntary basis. The purpose of the study was fully explained to patients and that recording was destroyed after transcription. They were also informed that the data would be anonymous. On accepting participation in the study, the informant signed an informed consent. The study was carried out in accordance with the recommendations in the Declaration of Helsinki II and approved by the Regional Ethics Committee (Assemblyn.d.). Research Ethics Committee approval for the project was not deemed necessary from the Regional Committee for Medical Research Ethics, because the project was not designated a medical research study. Approval from the Danish Social Science Data Services was obtained (VD‐2018‐136; I‐Suite nr.: 6379).

## 6 FINDINGS AND DISCUSSION

Our findings are presented as themes that emerged from the analysis. The presentation is a summation of the analysis and extracted interpretational segments, which revealed the themes that are consistent in the stories. The themes are expressions of what the informants expressed and include direct citations from the interviewees.

The overall theme that arose from the analysis was: “A confidential moment with the nurse to deal with and become more aware of what is important.” The theme consisted of three subthemes: “An option that makes sense,” “Scheduled time with the nurse is important” and “To find a new normality in everyday life.”

### 6.1 An option that makes sense

In this study, the informants ask for time with HCP to talk about health and the opportunity to ask questions. One of the informants said: “…. it is so nice that you want to implement nursing consultations … there has never ever been anyone who had the time for a dialogue..”. A systematic review found that patients asked for the special competences that nurses have. Nurses can get the patients to tell their story and to reflect and analyse it, and nurses provide longer consultations and more information to patients than physicians (Horrocks, Anderson, & Salisbury, 2002; Laurant et al., 2005, 2018) and that is what the informants in this study asked for. An informant explained it like this: “ ..it (the nursing consultation) is a very good idea if you only have the family to talk to…”. That is in line with findings showing that nurses can conduct the consultations and that patients are more open to nurses and will describe their problems and challenges from everyday life (Kinnersley et al., 2000). Some of the informants also mentioned that it was easier to talk to a nurse as: “you speak a more understandable language – and you can explain the difficult ones to me.” As most patients with chronic diseases live with daily challenges related to complex treatments, it is important to understand advice and instructions related to these treatments to stay as healthy as possible (Parker & Gazmararian, 2003). An informant stated it like this “you need to be responsible for your own health.” The nursing consultation might open with a dialogue, presenting thoughts and doubts about compliance and non‐compliance related to recommended guidelines (Ivarsson et al., 2013) or with nurses meeting patients who are interested in understanding their health predicaments and what can be done about them (Jonsdottir, Litchfield, & Pharris, 2004).

All the informants were very satisfied with the opportunity to be invited to attend a nursing consultation, though some of them expressed no actual need to attend one themselves but were sure that many other patients would benefit from attending a nursing consultation. It was observed that some of the informants who said they had no actual need were the ones who continued the dialogue, talking about a lot of challenges in everyday life such as decreased functional capacity. One of these informants said: “We are going to move to an apartment – I cannot handle our house any more.” As is well known, patients with chronic diseases do have complications related to their disease and treatment and neglecting symptoms could be interpreted as a way of pushing complications away. Defence mechanisms are psychological strategies that are unconsciously used to protect a person from anxiety arising from unacceptable thoughts or feelings (McLeod, 2018). Using a defence mechanism could be the informant's way of keeping the problems invisible and that could be the reason why the informant does not regard himself as having the need for a nursing consultation. This picture reflects the paradox that Parse mentions when she talks about making the invisible visible (Parse, 1998).

This study finds that informants have different expectations of nursing consultations. First, most informants highlighted the fact that the HCP was a nurse. Furthermore, some of the informants appreciated that the nurse was a woman. One of the informants said: “… it is very nice to talk to Dr. Smith [physician name] he is such a nice man, but there will always be something you do not tell the physician ‐ but do tell the nurse – a woman…”. The informants appreciated both the professional competences and the gender, but they did have different expectations related to gender. Stereotypes about the way men and women think and behave do have an impact on the way men and women define themselves and are treated by others (Ellemers, 2018). Men are characterized by assertiveness and performance and prioritization of work and career, while women are characterized by warmth and care and prioritize connectedness and family (Ellemers, 2018). The desire for interest and empathy could be the reason that the informants prefer to be met by a female nurse. In relation to the professional aspect, this perspective has been shown previously (Redsell, Stokes, Jackson, Hastings, & Baker, 2007).

### 6.2 Scheduled time with the nurse is important

The chosen framework for the nursing consultation was met as a positive setting for reflection by the participants even though some of the informants initially declared that they had no need for a consultation. Scheduled time seemed to be important to the informants. It was essential for the informant to know that the nursing consultation had been scheduled: “... if the nursing consultation is scheduled … then I will have planned the themes I want to talk about…”. It is obvious that this informant wanted to be prepared. Another informant described the issue about time like this: “...if I know what I want to reflect on....I perhaps do not need to talk to you for half an hour.” The informants knew that time could be crucial in a hospital setting—and in their own life too. They experienced that physicians were busy, with only 15 min planned per patient, so informants did not want to disturb the physician by asking questions, which, in their opinion, were not related to the treatment (Redsell et al., 2007). Contrary to the consultation with the physician, the time and the topic of the nursing consultation belonged to the patient because it was scheduled. The informants highlighted the opportunity to reflect on the topics using the past, present and future. Overseeing the nursing consultation gave the informants an experience of power and ownership and of being met as the persons they were, which could be interpreted as being in a place of freedom: “..Nursing consultations will be a good idea … there will always be something hidden, which will come forward.” Avoiding telling the physician about daily challenges could also be interpreted as a defence mechanism used by the informants to make problems invisible and to avoid facing them (McLeod, 2018). If a functional decrease is not diagnosed, the consequences are still invisible.

### 6.3 To find a new normality in everyday life

After lung transplantation, patients must adapt to a new normality, accepting the current condition and the limitations, which are facilitated by comparing the present condition to life pre‐transplant. As time goes by the patients also compare their present condition to their health status in earlier years as a person with a transplant living with a chronic disease (Lundmark, Lennerling, & Forsberg, 2019). This is comparable with conditions other patients with chronic diseases experience when there is a progression in their disease.

The informants in this pilot study described their new normality with different perspectives regarding age, gender, life experiences and health status. An informant said: “People tell me that I have got a new life. That is not true. I have got my life back – this time it was not just a tablet helping me out.” It is important to acknowledge that the person is still the same person despite being treated bodily to survive. One of the informants described the wish to be in control of his new normality of everyday life: “I live my life as I want to. I just hope for help from the physicians to live it.” Though people strive to find a new normality, basically people also want to be like other people even if they have to live with a chronic disease: “I do have a life to live. I do not have time to sit down and whine.” People strive to affiliate with other people even though they do not have the resources to do it (Lakin, Chartrand, & Arkin, 2008).

In some cases, the informants mentioned the importance of attending a nursing consultation with the nurse—alone: “…it is not necessary that everybody knows everything …. it might be something personally ‐ right?”. The informants wanted to discuss personal topics, which they did not want to be mentioned outside the nursing consultation. From the informant perspective, these discussions could be needed because they were required to protect the family from unnecessary worries.

To finish the nursing consultation, the informant and the nurse summarized the discussion together, making an agreement about what should be documented in the informant's journal.

### 6.4 Acceptability and applicability

None of the invited patients refused to participate in the nursing consultations. Instead, they were interested and positive related to the opportunity to attend the nursing consultations.

#### 6.4.1 Trustworthiness

In the qualitative methodology, the following criteria were used to assess the pilot study's trustworthiness: *credibility, dependability and transferability* (Graneheim & Lundman, 2004).

##### Credibility

In this paper, relevant information about background, methodology, methods, processes and analyses is presented. The informants were the patients, who came for the annual check‐up. They would have some experience living with a chronic disease. The statements of the informants were repeated to them during the interview to ensure that the interpretation was correct. Furthermore, the authors read through the transcribed text several times to increase the ability to explore the informants’ experiences and both conducted the analysis separately. All interviews were conducted at the hospital and were scheduled just after the nursing consultation.

##### Dependability

In this pilot study, data were collected in two months and the authors collected data together. The authors are aware that interviewing and observing is an evolving process and have discussed the progress of the interviewer.

##### Transferability

To ensure an appropriate assessment of transferability, relevant information, for example demographic data and disease‐related characteristics of the informants, was presented, as were the inclusion criteria. Appropriate quotations will throw light on how the findings can be transferred to other groups of patients with chronic diseases and their need for an opportunity to have a personal talk with a HCP.

## 7 CONCLUSION

The pilot study showed acceptance and applicability to the possibility of attending nursing consultations for patients with advanced heart and lung failure. The informants saw the intervention as useful and meaningful, related to having an opportunity to reflect on everyday challenges. The framework inspired by Parse addressed a space of freedom requested by the informants. This pilot study highlighted the informants’ need for “A confidential moment with the nurse to deal with and become more aware of what is important.”

## CONFLICT OF INTEREST

None to declare.

## AUTHOR CONTRIBUTION

Study design: JG conceived the idea for this study. JG and IEH designed the study and wrote together the first draft of the manuscript. Both authors revised the manuscript critically and have given their final of the version to be published.

## RELEVANCE TO CLINICAL PRACTICE

This pilot study indicates that patients with advanced heart and lung failure consider it important to have an opportunity to talk to and reflect with a nurse about topics of importance to them. The reason why they preferred to talk to a healthcare professional instead of family and friends was to reduce the caregiver burden. This knowledge is important because it does prove the need for scheduled time with a healthcare professional to reduce everyday challenges and thereby improve quality of life. The indication for further research will be to explore the content of nursing consultations to get insight in everyday phenomena of patients living with advanced heart and lung failure.
